# Decoding antibacterial and antibiofilm properties of cinnamon and cardamom essential oils: a combined molecular docking and experimental study

**DOI:** 10.1186/s13568-021-01305-6

**Published:** 2021-10-26

**Authors:** Elahe Pourkhosravani, Fatemeh Dehghan Nayeri, Mitra Mohammadi Bazargani

**Affiliations:** 1 Agricultural Biotechnology Department, Faculty of Agriculture and Natural Sciences, Imam Khomeini International University (IKIU), Qazvin, Iran; 2 Medicinal Plant Department, Agriculture Institute (ARI), Iranian Research Organization for Science and Technology (IROST), Tehran, Iran

**Keywords:** Antibiofilm, Bacteria, *Cinnamomum verum*, *Elettaria cardamomum*, Essential oil

## Abstract

This study sets out to compare the antibacterial and antibiofilm profiles of Ci/Ca EOs alone and in combination together against infectious bacterial strains. MIC assay was carried out to survey the effectiveness of prepared EOs by two-fold serial dilution method and MTT evaluation. Synergic antibacterial properties of EOs against target strains were studied by using checkerboard titration method. Biofilm growth and development were evaluated using CV and XTT reduction assays. Antibacterial activity was observed for EOs against both bacterial strains with stronger activity for CiEO against both bacteria. The synergistic antibacterial effect was observed only against *B. subtilis*. Based on the FIC index, combinations could not inhibit the growth of *E. coli*. The pure EOs and their combination inhibited cell attachment for both studied bacteria with stronger effect on *E. coli*. CV and XTT reduction assays results showed that Ci EO and its combination with CaEO had the highest antibiofilm activity at lowest MIC value 0.08% and 0.04/0.02% against biofilm formed by *E. coli* and *B. subtilis* respectively, indicating a high antibiofilm potential. Computational docking analyses also postulated that the active constituents of evaluated EOs have the potential to interact with different bacterial targets, suggested binding mode of action of EOs metabolites. By and large, synergistic anti-biofilm properties of EOs may provide further options for developing novel formula to inhibit a variety of infectious clinical and industrial strains without (or less) toxicity effects on human body.

**Supplementary Information:**

The online version contains supplementary material available at 10.1186/s13568-021-01305-6.

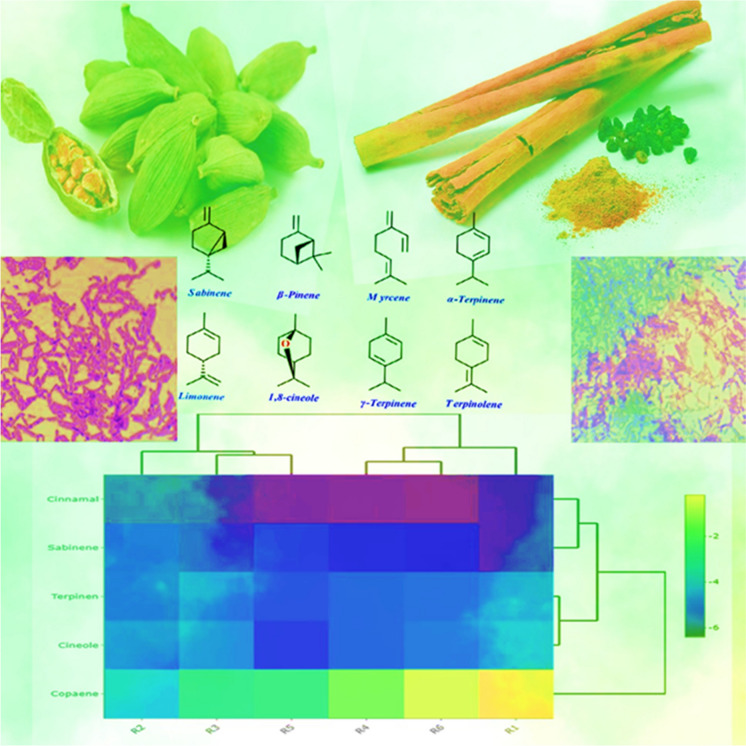

## Key points

- The Ci/Ca EOs combination had the lowest antibiofilm activity against *E. coli* compared with pure Ci and Ca EOs.
- The Ci/Ca EOs combination showed synergistic antibacterial effect against *B. subtilis.*
- The metabolites of two EOs interact with different bacterial targets with moderate to low binding energies.

## Introduction

Today, finding promising natural and synthetic agents with potential anti-bacterial activity is becoming so popular among scientists. A variety of plant extracts (or essential oils) and chemical substances have been evaluated to identify their toxicity profiles on infectious organisms (Zarayneh et al. 2018). Despite all efforts in this field, the accumulating body of evidence suggests that only a few numbers of practical antibacterial agents commercially available to fight against infectious strains (Loolaie et al. 2017).

Biofilm is a mixture of various groups of microorganisms in a complex with extracellular polymer matrix in which cells bind tightly together on various surfaces (Wang et al. 2020). Biofilm usually creates a sticky gel composed of polysaccharides, proteins and other organic components on a wet surface, that occur in different clinical, industrial and food processing environments and cause serious problems including cross contamination of food products (Muslim et al. 2018). Bacterial biofilms are inherently more tolerant to antibiotic therapies and chemical agents compared with planktonic cells in suspension (Felipe et al. 2019).

According to the review of literature, *Escherichia coli,* a gram-negative bacterium, is one of the most versatile microorganisms reported in nature, contributes to form biofilm easily on the different surfaces of food industry (Frozi et al. 2017). Similarly, *Bacillus subtilis* is a gram-positive soil bacterium that is constantly exposed to a broad range of environmental stresses (Borriss et al. 2018) and can respond to stressful conditions by forming biofilm on different surfaces (Romero et al. 2010). With resistance to a variety of external stresses, the biofilm formed by *B. subtilis* may cause serious medical problems by making particular wounds difficult to treat, contaminating implants and other medical devices and causing surface corruption in food industries (Ryu and Beuchat 2005).

Over the past decades, EOs reached a great level of academic interest among researchers because of their effectiveness for controlling a variety of infectious organisms. Considerable number of studies have tested EOs biological properties for inhibition of *E. coli* and similar strains. Results indicated that EOs from tea tree, lavender, and lemon balm oil (Budzyńska et al. 2011), cinnamon (*Cinnamomum verum* L.) showed synergistic effects on biofilm formation of several different bacterial strains. Nevertheless, limited information exists regarding the antibiofilm activity of plant extract or essential oil against *B. subtilis* (Chemsa et al. 2018). The EOs of *C. verum* L. and cardamom (*Elettaria cardamomum* L.) display antibacterial activity against both Gram-positive and negative strains*.* These two plants are among most appreciated medicinal plants in Asian countries, in particular India and Iran, because of their health benefits, antioxidant properties and potential to alleviate gastrointestinal pains (Chemsa et al. 2018; Jamal et al. 2006). However, while practical health benefits of these plants are well understood, there is no report about the antibacterial and antibiofilm activities for the combinatorial effect of the cinnamon/cardamom EOs mixture against *E. coli* and *B. subtilis* and also there is a little bit information regarding molecular mode of action of their biological active compounds.

Recent progresses on computational assays for unraveling the possible interaction of natural products and cellular receptors provided a new way to easily predict the molecular action of plant secondary metabolites. Among applied computational methods, docking analysis is one of the most important and handy methods because of its simple application to predict the inhibitory mode of action of chemical ligands against their favorable receptors (Rasouli et al. 2017, 2020). It is not exaggerating if we say at least half one-third of our knowledge regarding the inhibitory properties of EOs and purified metabolites against a variety of receptors directly generated from docking analyses.

Therefore, the aim of this study was to investigate the antibacterial and antibiofilm activities of cinnamon and cardamom essential oils against *E. coli* and *B. subtilis* alone and in combination using in vitro assays. Furthermore, we coupled docking analysis and experimental procedure to show how EOs may inhibit the biofilm formation of infectious bacterial strains that presently causing a variety of problems for both clinics and food industries.

## Material and methods

### Plant material and extraction

EOs were prepared by hydro-distillation method from the seed of cardamom and the trunk bark of cinnamon using a Clevenger-type apparatus for 3.5 h (British Pharmacopoeia, 1998). All prepared samples were stored in the dark at 4 °C for further analysis.

### Bacterial strains

Both Gram-positive *B. subtilis* (NCTC 5398) and Gram-negative *E. coli* (DH5alpha) bacteria were provided by biotechnology laboratory of Imam Khomeini International University (IKIU).

### Determination of minimum inhibitory concentration (MIC)

MIC analysis was conducted by using two-fold serial dilution technique as described previously in (Bazargani and Rohloff 2016) in three replicates. The bacterial suspensions were allotted to a concentration approximately 10^6^ CFU/ml in (Pandey et al. 2011; Sandasi et al. 2010). In all assays to improve the oil solubility, MHB supplemented with 2% (v/v) DMSO was used (Silva et al. 2011). Each EO was serially diluted in MHB in a 96-well microtiter plate to have a final EO concentration ranging from 2.5 to 0.02% (v/v). Then 100 µl of bacterial suspension was inoculated to each well and the plate incubated at 37 °C for 24 h (Rangasamy et al. 2007; Sarker et al. 2007). To enhance the accuracy of all assays, positive and negative controls were ciprofloxacin (1 mg/ml), (used instead of plant extract) and DMSO + MHB (applied instead of bacterial solution), respectively (Sandasi et al. 2010). Finally, MTT (3-(4,5-dimethyl-2-thiazolyl)-2,5-diphenyl-2H tetrazolium bromide) solution (0.2 mg/ml) was added to each well and the plate, then incubated at room temperature for 10 to 15 min. The lowest concentration of EO with no visible pink-red coloration was recorded as the MIC value.

### Determination of fractional inhibitory concentration index (FICI)

To evaluate the efficacy of possible interaction between EOs against both bacteria strains, serial two-fold dilutions of cinnamon (2.5–0.02% v/v) and cardamom (2.5–0.02% v/v) EOs were mixed as described in (Fei et al. 2011) and applied on both bacteria with a similar method adopted to assess MIC. Subsequently, 100 µl of bacterial suspension was inoculated to each well in a microtiter plate and the plate was incubated at 37 °C for 24 h. The growth condition was the same as previously used for MIC assay. FIC index (FICI) was calculated using the following formula: FICI = FIC_A_ (MIC of EOA in combination with EOB/ MIC of EOA alone) + FIC_B_ (MIC of EOB in combination with EOA/ MIC of EOB alone), where EOA and EOB are two different essential oils and FIC_A_ and FIC_B_ are fractional inhibitory concentration of EOA and EOB respectively. To determine the type of interactions, the following thresholds were considered for FICI values: FICI ≤ 0.5 for synergistic; 0.5 < FICI ≤ 1 for additivity; 1 < FICI ≤ 4 for indifference or no effect; and FICI ≥ 4 for antagonistic effects (Fei et al. 2011; Gutierrez et al. 2009).

### Inhibition of initial bacteria cell attachment

The effect of pure EOs and their combinations on inhibition of initial bacteria cell attachment was determined as a procedure described in (Bazargani and Rohloff 2016). The EOs with an amount similar to their MIC value were added to each well in a 96-well microtiter plate. Ciprofloxacin (MIC value) and MHB were used as positive and negative controls, respectively. Bacteria culture (10^6^ CFU/ml) was inoculated into each well. Also, only MHB was added to blank wells. In order to precede the attachment of cells on the surface, the plate incubated at 37 °C for 8 h without any shaking. After incubation and removing the contents of each wells, plates were washed with sterile distilled water for three times and placed at 60 °C for 45 min. To perform the crystal violet staining assay, the plate was stained with 1% crystal violet, incubated at room temperature for 15 min, rinsed three times by using sterile distilled water then de-stained with ethanol. Finally, 100 μl of the de-stained solution was transferred to a new plate and the absorbance recorded in three replicates at OD_590 nm_ using a microplate ELISA reader (Bio Tek Instruments, USA). The percentage inhibition was calculated with the following formula (Sandasi et al. 2010):

$${\text{Percentage}}\;{\text{inhibition}} = \frac{{{\text{OD}}_{{\text{Negative control}}} {-}{\text{ OD}}_{{{\text{Experimental}}}} }}{{{\text{OD}}_{{\text{Negative control}}} }} \times 100.$$

### Inhibition of biofilm formation and development—biofilm biomass measurement

The effect of plant EOs and their combinatorial effects to inhibit the biofilm formation and development was determined with a procedure described in Bazargani and Rohloff (2016). After the biofilm formation for 4 h at 37 °C, plant EO was added to yield a final concentration similar to their respective MIC values and the mixture incubated for 24 h. Crystal violet staining assay was conducted as described in previous section to determine the inhibition of biofilm growth and development.

### Biofilm metabolic activity measurement

The metabolic (respiratory) activity of biofilm was determined by using (XTT) reduction assay as described in Bazargani and Rohloff (2016). After mixing the plant EO with biofilm and incubation for 24 h at 37 °C, the liquid part from each well was removed and each well washed three times with phosphate-buffered saline (PBS). The sodium salt of XTT (2,3-bis(2-methoxy-4-nitro-5-sulfophenyl)-2H-tetrazolium-5-carboxanilide) was dissolved in PBS at a final concentration of 1 mg/ml, filter-sterilized and stored at 80 °C. Menadione was dissolved in acetone to 1 mM and sterilized immediately before each measurement. Then, 100 µl PBS was added in each well, and then 13.5 µl of fresh XTT/menadione mixture (12.5:1) was mixed in each well. The microtiter plate was gently shaken, covered with aluminum foil and incubated at 37 °C for 2 to 3 h, then absorbance was read in three replicates at 490 nm (Pettit et al. 2005). The negative and positive controls and blank wells were prepared as described previously (Chaieb et al. 2011).

### Gas chromatography-mass spectrometry (GC/MS)

The chemical components of EOs were determined by GC–MS. EO samples were diluted in hexane (5 μl/ml)), injected in a split mode at a ratio of 1:35 and analysed using an Agilent HP-6890 Gas Chromatograph coupled with Agilent HP-5973 mass selective detector equipped with a BPX5 fused silica column (30 m × 0.25 mm ID, 0.25 μm film thickness). The GC column temperature was programmed from 50 to 300 °C via a ramp of 3 °C/min, and maintained at 300 °C for additional 3 min. The carrier gas was helium with a flow rate of 0.5 ml/min. The MS source was adjusted to 220 °C, and a mass range of m/z 40–500 was recorded acquiring all mass spectra in EI mode. The chromatogram visualization and determining the peak area integration was performed with Agilent Chemstation software (Agilent Technologies, Waldbronn, Germany). EOs chemical constituents were identified based on search in MS database using Mass Spectral Library (NIST 05), an in-house retention index library of MS spectra of volatile organic compounds, and comparison of spectra with MS data reported in literature (Sparkman 2005).

### Microscopic visualization of biofilm

The effect of plant EOs on inhibition of biofilm formation was visualized with microscopic as described in Bazargani and Rohloff (2016). The biofilm formation (as described above) was performed on round cover glass slides (diameter 1 cm) placed in 24-well plate (Greiner Bio-One, France). The pure and combinatorial EOs with an amount similar to MIC value with high and low antibiofilm activity against bacteria were added to the developed biofilm. The negative and positive controls were also prepared as described above and the plate incubated for 24 h at 37 °C. The glass slides were stained with 1/20 (v/v) Giemsa (Sigma, Switzerland) solution for 20 min at room temperature. For different treatments, biofilm formation was visualized using a light microscope with 100 × magnification (Bazargani and Rohloff 2016; Chaieb et al. 2011).

### Computational details

To show the binding mode of action of active compounds isolated from both plants using GC/MS procedure, docking analysis were performed. First, the “.*sdf*” format of identified ligands were created using Gaussian 03 software and then converted into “*.pdb*” formats. All prepared ligands were optimized using the above-mentioned software for docking analysis. According to GC/MS output, most of identified compounds in these plants were terpenes and their chemical backbone were selected as input chemical ligands for generating docking poses. To perform docking analysis, we used AutoDock Tools and vina scripts. Receptors were chosen among proteins that critically are important for biofilm formation. In this regard, X-ray crystallographic structure of proteins including Bap1 (pdb id: IND1), FabH (pdb id: 1HN9), FtsZ (pdb id: 2VXY), MerB (pdb id: 2WUS), RbmC (pdb id: 5V6C) and RbmA (pdb id: 4KKP) were obtained from PDB database. The receptors were checked for unfavorable atoms, and all water molecules were deleted within the downloaded *pdb* files. After that, polar hydrogens and surface charges were added to these receptors and their main backbone were selected for docking analysis. UCSF chimera was used for visualizing the docked poses and all docking steps were checked for possible errors and producing false-positive results.

## Results

### Determination of minimum inhibitory concentration (MIC)

**Fig. 1:**
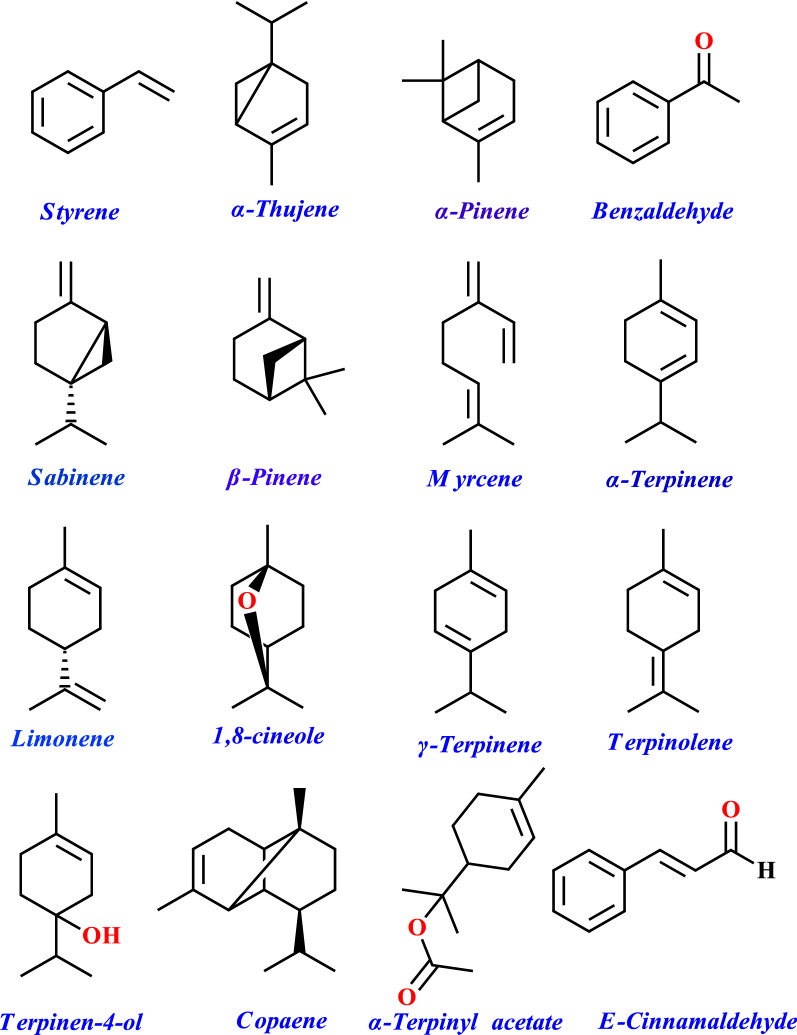
2D illustration of most frequent chemical compounds isolated from both Eos

Antimicrobial activities of EOs of *E. cardamomum* L. and *C. verum* L. displayed the highest antibacterial activity with MIC values of 0.08% and 0.31% (v/v) against *E. coli* and *B. subtilis*, respectively (Table 1). Figure 1 also represents 2D graphical illustration of frequent chemical constituents isolated from studied EOs.

**Table 1:** MIC values concentration of cardamom and cinnamon essential oils against *E. coli* and *B. subtilis.* MIC were evaluated after 24 h of incubation

Concentration (%v/v)	*E. coli*		*B. subtilis*	
	Cardamom	Cinnamon	Cardamom	Cinnamon
2.5	−	−	−	−
1.25	+	−	+	−
0.63	+	−	+	−
0.31	+	−	+	−
0.16	+	−	+	+
0.08	+	−	+	+
0.04	+	+	+	+
0.02	+	+	+	+

+ : Presence of growth; −: absence of growth

### The combinatorial assay

The FIC values for the combinatorial antibacterial effect ranged from 0.03% to 2% and 0.01% to 0.13% against *E. coli* and *B. subtilis,* respectively (Table 2). A synergistic effect (FIC index < 0.5) was observed only against *B. subtilis* for two combinations, 1/64 MIC of cardamom + 1/8 MIC of cinnamon and 1/128 MIC of cardamom + 1/8 MIC of cinnamon with a FIC index of 0.15 and 0.14, respectively. However, the FIC index for combinatorial effect with 1/64 MIC of cardamom + 1 MIC of cinnamon and 1/64 MIC of cardamom + 1/2 MIC of cinnamon with no inhibition effect on *E. coli* growth was 1.02 (indifferent) and 0.52 (additive), respectively (Table 2).

**Table 2:** FIC, FIC index and the results of interaction of cardamom and cinnamon essential oils combinations against *E. coli* and *B. subtilis*

Bacteria strain	Plant EO	MIC (% v/v)		FIC (%v/v)	FICI	Remarks
		Alone	Combination			
*E. coli*	Cardamom	2.5	0.04	0.02	0.52	Additive
	Cinnamon	0.08	0.04	0.50		
	Cardamom	2.5	0.04	0.02	1.02	Indifferent
	Cinnamon	0.08	0.08	1		
*B. subtilis*	Cardamom	2.5	0.04	0.02	0.15	Synergist
	Cinnamon	0.31	0.04	0.13		
	Cardamom	2.5	0.02	0.01	0.14	Synergist
	Cinnamon	0.31	0.04	0.13		

### Inhibition of initial bacteria cell attachment (biofilm inhibition)

The results of anti-adhesion tests conducted with the crystal violet assay showed that CiEO inhibited cell attachment of *E. coli* completely (100% inhibition activity), while CaEO and their combination (1/64 MIC of cardamom + 1/2 MIC of cinnamon) displayed 92% and 62% inhibition against *E. coli,* respectively (Table 3). Moderate inhibition was observed for EOs against *B. subtilis* and their combination against *E. coli* (Table 3). *B. subtilis* was more resistant than *E. coli* with a lower inhibition, 54% and 76% for Ci/Ca EOs, respectively. However, the combination 1/128 MIC of cardamom + 1/8 MIC of cinnamon showed more inhibition, 80% compared with pure EOs against *B. subtilis* (Table 3).

**Table 3:** Antibiofilm activity of cardamom and cinnamon essential oils and their combination on inhibition of initial bacteria cell attachment and biofilm formation (growth and development)

Plant EO	Strain	MIC value (%v/v) Concentration	% Inhibition of bacteria attachment	% Inhibition of biofilm formation	
			Crystal violet	Crystal violet	XTT
Cardamom	*E. coli*	2.5	92	68	57
Cinnamon		0.08	100	100	99
Cardamom/Cinnamon		0.04/0.04	62	58	61
Cardamom	*B. subtilis*	2.5	76	41	33
Cinnamon		0.63	54	55	36
Cardamom/Cinnamon		0.02/0.04	80	98	58

In general, the amount of inhibition of biofilm formation was less pronounced compared to inhibition of initial attachment except for CiEO which showed the same inhibitory activity at attachment and biofilm formation against both bacteria. Also, Ci/Ca EOs combination showed 98% inhibitory biofilm formation activity at MIC value of 0.02/0.04% (v/v) against *B. subtilis* which was more observed compared with inhibition of initial attachment (Table 3).

The reduced inhibition of biofilm development demonstrated that the bacteria cells in a biofilm are more resistant to antimicrobial agents compared to planktonic cells. In fact, inhibition of biofilm growth and development is more difficult to achieve than inhibition of cell attachment. These results are consistent with the results of previous studies (Bazargani and Rohloff 2016; Sandasi et al. 2008). Based on the findings of the present study, CiEO and Ci/Ca EOs mixture exhibited the highest inhibitory biofilm formation activity against *E. coli* and *B. subtilis* respectively with lowest MIC values (0.08% and 0.02/0.04% v/v) (Table 3). It has been reported that oregano oil at 0.1% (v/v) inhibited biofilm formation of *E. coli* (Oral et al. 2010). The *Cinnamomum zeylanicum* EO inhibited biofilm formation at inhibitory concentration of 1.92% (v/v) which was the most satisfactorily with *E. coli* reduction of biomass (Millezi et al. 2019). The essential oil of another cinnamon species (*Cinnamomum burmannii*) was able to inhibit biofilm formation of *S. aureus* and *P. aeruginosa* by 50% at concentration of 0.03% (v/v) and destabilized biofilms of both bacteria at a concentration of 0.12% (v/v) (Pratiwi et al. 2015). Other studies have reported effectively removal of *E. coli* and *S. epidermidis* biofilms by CiEO (de Oliveira et al. 2012; Nuryastuti et al. 2009). Interestingly, Oh et al (2017) evaluated the effects of single and blended essential oils of thymol, oregano and cavacrol on biofilm formation of *Salmonella* and *E. coli*. They observed single essential oil had a better antibiofilm property than blended essential oil (Oh et al. 2017). In that study, thymol and oregano EOs had a better antibiofilm activity against *E. coli* than cavacrol EO and in comparison to oregano EO, both cavacrol and thymol EOs had a better antibiofilm effect on *Salmonella* (Oh et al. 2017). Similar to previous investigations, the current study showed CiEO had a more antibiofilm activity against *E. coli* bacterial biofilm than cardamom and Ci/Ca EOs mixture. Nevertheless, Ci/Ca EOs combination indicated more percentage inhibition of *B. subtilis* biofilm formation than single essential oil which may be related to synergistic antibacterial effect of blended EOs against this bacterium.

### Inhibition of biofilm formation-biomass quantification (crystal violet assay)

The results of quantification of biofilm biomass revealed that EO of cinnamon inhibited biofilm formation of *E. coli* completely (100% inhibition activity) while EO of cardamom and the combination 1/64 MIC of cardamom + 1/2 MIC of cinnamon induced inhibition of biofilm formation against *E. coli* up to 68% and 58%, respectively (Table 3). In comparison, EO of cinnamon displayed a low biofilm inhibition (55%) against *B. subtilis*. Similarly, EO of cardamom exhibited 41% inhibition activity against biofilm formed by *B. subtilis* whereas their combination, 1/128 MIC of cardamom + 1/8 MIC of cinnamon showed a high inhibition (98%) and thus a strong antibiofilm activity compared with pure EOs against *B. subtilis* (Table 3). Moreover, *B. subtilis* showed more resistance to pure EO than *E. coli*.

### Inhibition of biofilm formation-metabolic activity (XTT assay)

The results of evaluation metabolic (respiratory) activity of biofilm indicated that pure EOs and their mixture decreased metabolic activity of bacterial cells in biofilms formed by *E. coli* and *B. subtilis*, with 57 to 99% and 33 to 58% inhibition, respectively (Table 3). CiEO was most effective to inhibit the biofilm formation of *E. coli* by 100% indicating decomposing and degrading the biofilm completely. Our data also provided evidence that CiEO had the highest inhibitory potential in which could reduce metabolic activity of *E. coli* by 99%. The EOs mixture didn’t have any more reduction compared to pure EOs. Ci/Ca EOs displayed a lower reduction (36% and 33%, respectively) in metabolic activity against *B. subtilis* biofilm compared to *E. coli.* The EOs mixture (1/128 MIC of cardamom + 1/8 MIC of cinnamon) showed more reduction (58%) in metabolic activity against *B. subtilis* (Table 3). *B. subtilis* had a more resistance to reduction in metabolic activity by using pure EO than *E. coli* (Table 3). Moreover, the results of anti-adhesion, crystal violet and XTT assays revealed that the pure CiEO (at MIC value of 0.08%) and in combination with CaEO (with MIC value of 0.02 and 0.04% respectively) were effective in reduction of biofilm biomass and also in reducing metabolic activity of biofilm formed by *E. coli* and *B. subtilis*.

As shown in Table 3, the biomass production and metabolic activity assay were almost identical. Moreover, the inhibition of biofilm formation by both EOs was also confirmed by XTT reduction assay. With the exception of CiEO combination with CaEO used against *B. subtilis*, which the result of XTT reduction assay was not correlated with crystal violet assay inhibition of biofilm formation (Table 3). Despite of the increased inhibitory effect of biofilm formation, the metabolic activity did not decrease significantly compared to the inhibited biofilm. In this regard, several studies indicated an inverse correlation or no correlation between biomass production and metabolic activity for plant extract or essential oil (Budzyńska et al. 2011; Kwieciński et al. 2009; Sandasi et al. 2008). Overall, the essential oil of cinnamon had the highest inhibitory effect on the biofilm growth against both tested bacteria particularly *E. coli* and the combination of Ci/Ca EOs had the best effect against *B. subtilis* biofilm.

### Chemical composition of essential oils

In total, 17 and 21 chemical compounds were identified with GC–MS analyses in cinnamon and cardamom, respectively (Table 4). Three main components including E-cinnamaldehyde, copaene and α-terpinyl acetate constituted 91.31% of the CiEO. In contrast, 1,8-cineole, α-terpinyl acetate, sabinene, and α-terpinen-4-ol were the main constituents (89.58%) of CaEO (Table 4). The total amount of terpenes in essential oil of cinnamon and cardamom was 11.51% and 98.36% respectively, of which 5.09% and 90.92% were oxygenated monoterpenes.

**Table 4:** Chemical composition (%) of cinnamon and cardamom essential oils

Compound	RI^a^	Cinnamon	Cardamom	Type of compound
Styrene	907	1.08	–^b^	Other
*α*-Thujene	938	–	0.13	MH
*α*-Pinene	946	–	1.06	MH
Benzaldehyde	986	0.43	–	Other
**Sabinene**	**988**	–	**3.06**	MH
*β*-Pinene	993	–	0.48	MH
Myrcene	1004	–	1.13	MH
*α*-Terpinene	1033	–	0.29	MH
Limonene	1044	0.13	–	MH
**1,8-cineole**	**1050**	1.22	**49.10**	MO
*γ*-Terpinene	1075	–	0.55	MH
*cis-*Sabinene hydrate	1089	–	0.38	MO
Terpinolene	1101	–	0.20	MH
Linalool	1118	–	0.47	MO
*trans*-Sabinene hydrate	1121	–	0.31	MO
**Terpinen-4-ol**	**1203**	–	**2.15**	MO
*α* –Terpineol	1219	0.09	1.57	MO
Linalyl acetate	1265	–	0.33	MO
Geraniol	1271	–	0.92	MO
Cinnamaldehyde	1278	0.48	0.42	Other
***E*-Cinnamaldehyde**	**1321**	**84.23**	–	Other
Methyl geranate	1342	–	0.15	MO
***α* -Terpinyl acetate**	**1370**	**3.51**	**35.54**	MO
**Copaene**	**1395**	**3.57**	–	SH
Caryophyllene	1441	0.25	–	SH
Acetic acid, cinnamyl ester	1477	0.26	–	Other
*α-*Amorphene	1497	0.24	–	SH
*β*-Selinene	1513	–	0.28	SH
*α*-Selinene	1514	0.11	–	SH
*α*-Muurolene	1522	0.83	–	SH
*δ*-Cadinene	1541	0.90	–	SH
Cadine-1,4-diene	1547	0.39	–	SH
Nerolidol	1581	–	0.26	SO
*α*-Cadinol	1675	0.27	–	MO
**Total identified (%)**		**97.99**	**98.77**	
**Monoterpenes (MT), total**		**5.22**	**97.82**	
*oxygenated MT*		5.09	90.92	
**Sesquiterpenes (ST), total**		**6.29**	0.54	
*oxygenated ST*		–	0.26	

*MH* monoterpene hydrocarbons, *MO* oxygenated monoterpenes, *SH* sesquiterpene hydrocarbons, *SO* oxygenated sesquiterpenes

^a^Kovats retention index. ^b^Below threshold level of < 0.01, or not detected, Levels of major compounds (≥ 2%) are marked in bold

### Microscopic visualization of biofilm formation

**Fig. 2:**
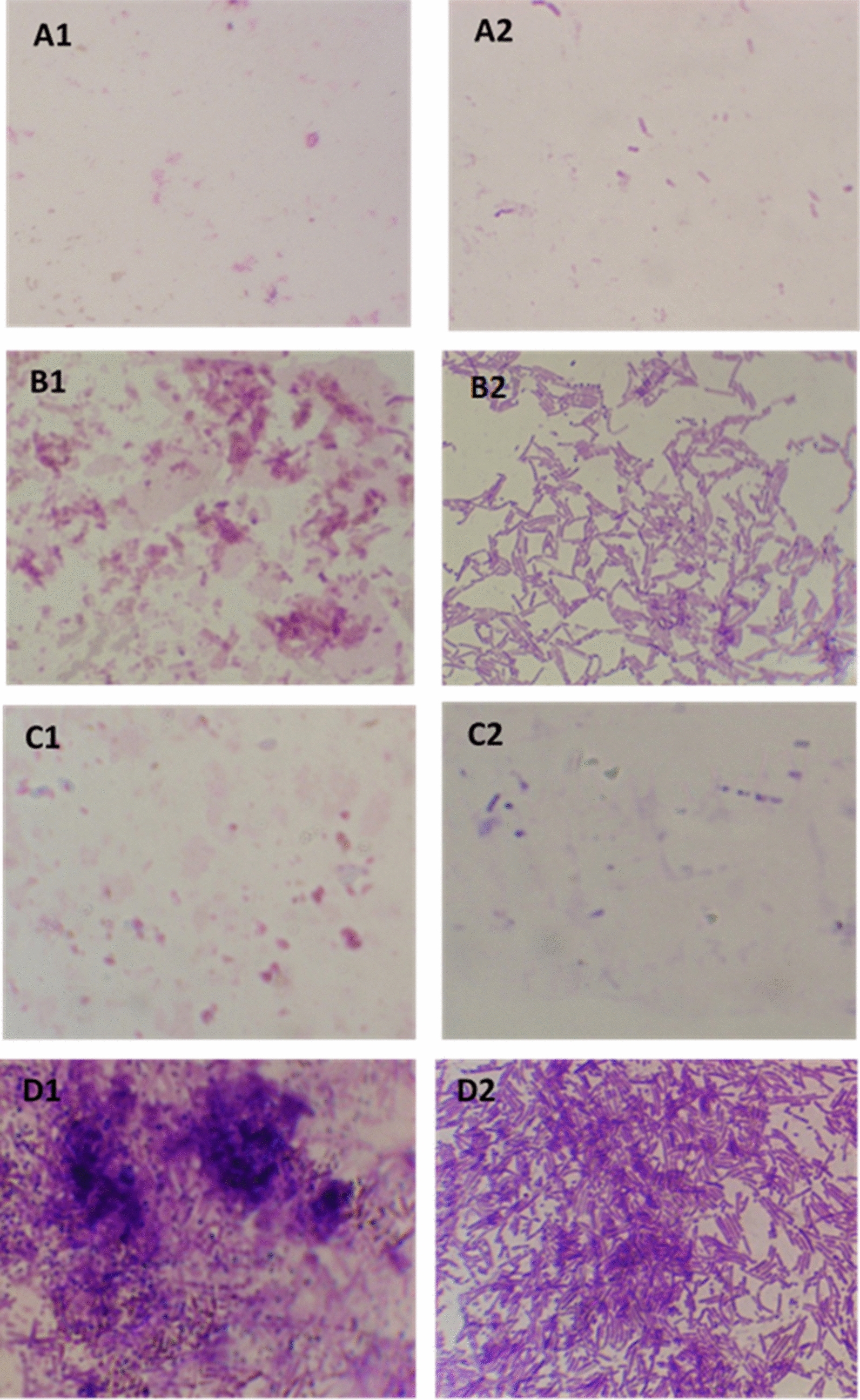
Light microscopy assay. Effect of essential oils (EO) on inhibition of biofilm formation (growth and development); *Escherichia coli,* **A1** bacteria supplemented with EO of cinnamon at MIC (high activity), **B1** bacteria supplemented with Ci/Ca EOs combination MIC (poor activity), **C1** positive control (bacteria supplemented with antibiotic MIC), **D1** negative control (non-treated slides). *Bacillus subtilis*, **A2** bacteria supplemented with EO of cinnamon/cardamom combination at MIC (high activity), **B2** bacteria supplemented with CaEO at MIC (poor activity), **C2** positive control (bacteria supplemented with antibiotic MIC), **D2** negative control (non-treated slides)

The high inhibition of biofilm formation by pure Ci EO and Ci/Ca EOs combination against *E. coli* and *B. subtilis* was also observed using microscopic visualization respectively (Fig. 2). The induction of biofilm growth and development was evident by CaEO and mixture of both EOs (with poor antibiofilm activity) against *B. subtilis* and *E. coli* respectively. The inhibition pattern of biofilm formation for ciprofloxacin (positive control) was similar to that observed for CiEO.

### Molecular docking outputs

**Fig. 3:**
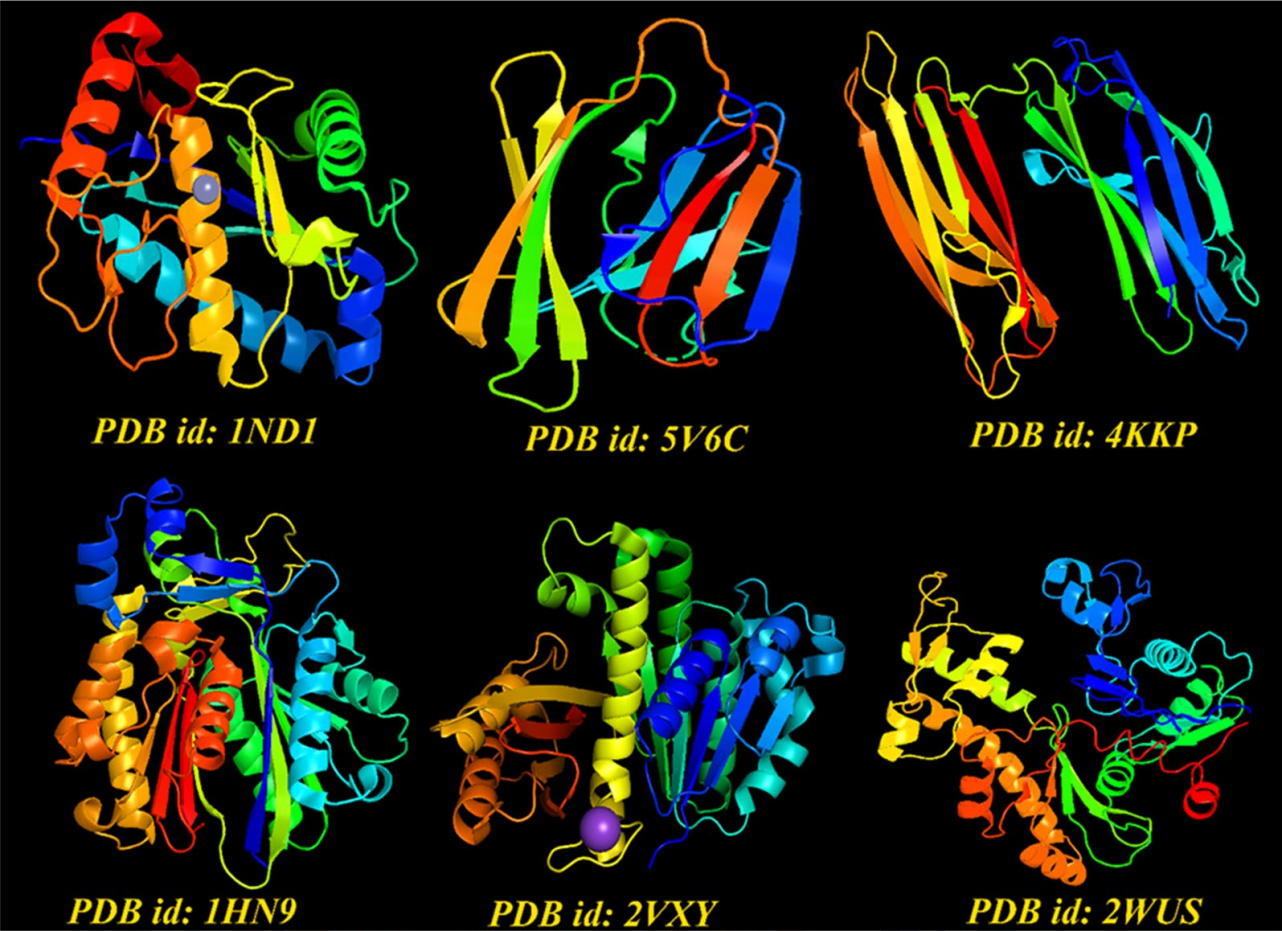
The graphical illustration of selected receptors for docking studies

Docking analysis is an excellent toolkit to support researchers for unravelling the exact molecular action of protein-ligands. In some cases, that access to experimental data is not provided, using docking strategy can pave a way to interpretate molecular aspects of cells. In this study, we used this method to determine how active constituents of CiEO and CaEO could biologically interact with molecular targets within bacterial biofilms. Six receptors (Fig. 3) were selected and binding mode inhibitory profile of these compounds were investigated.

**Fig. 4:**
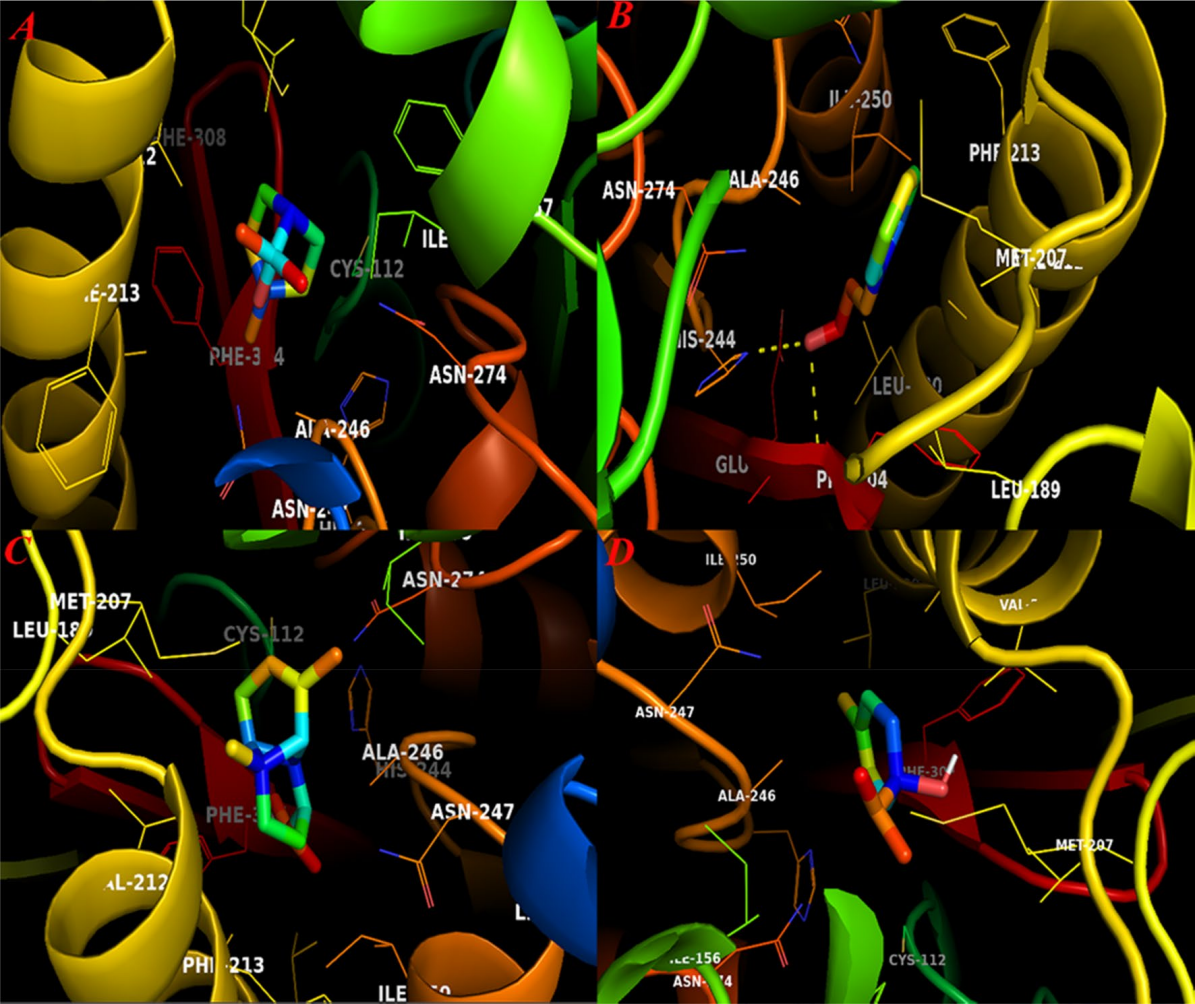
The graphical illustration of docked ligands and β-Ketoacyl-Acyl Carrier Protein Synthase III. **A** 1,8-cineole; **B** *E*-Cinnamaldehyde; **C** Copaene and **D** Terpinen-4-ol

As shown in Fig. 4, docked compounds exactly inserted within active site cavity. Although these compounds displayed a similar binding energy, nevertheless, *E*-cinnamaldehyde showed higher tendency to bind to catalytic residues in this active site. The binding docking energy for this compound was − 5.9 kcal mol^−1^. This compound was the only constituent that could form H-bonds with the active site of β-Ketoacyl-Acyl Carrier Protein Synthase III. Qiu et al. (2001) reported that several different amino acids including Cys112, Leu142, Phe157, Leu189, Asn274, Gly305 and Gly306 are critical residues in this cavity that modulate the functionality of enzyme in the presence of its substrate. Our docking results displayed that His244 and Phe304 were two residues that could build H-bond with the side chain of *E*-cinnamaldehyde metabolites. Among docked compounds for this receptor, copaene showed the lowest docking binding energy in which − 0.2 kcal mol^−1^ was its interaction energy. While sabinene possessed docking energy equals to − 5.9 kcal mol^−1^, this compound doesn’t build H-bond with possible catalytic residues in this active cite. 1,8-cieole and terpinene-4-ol compounds also inserted in this cavity and their docking energies were − 4.1 and − 4.3 kcal mol^−1^, respectively.

**Fig. 5:**
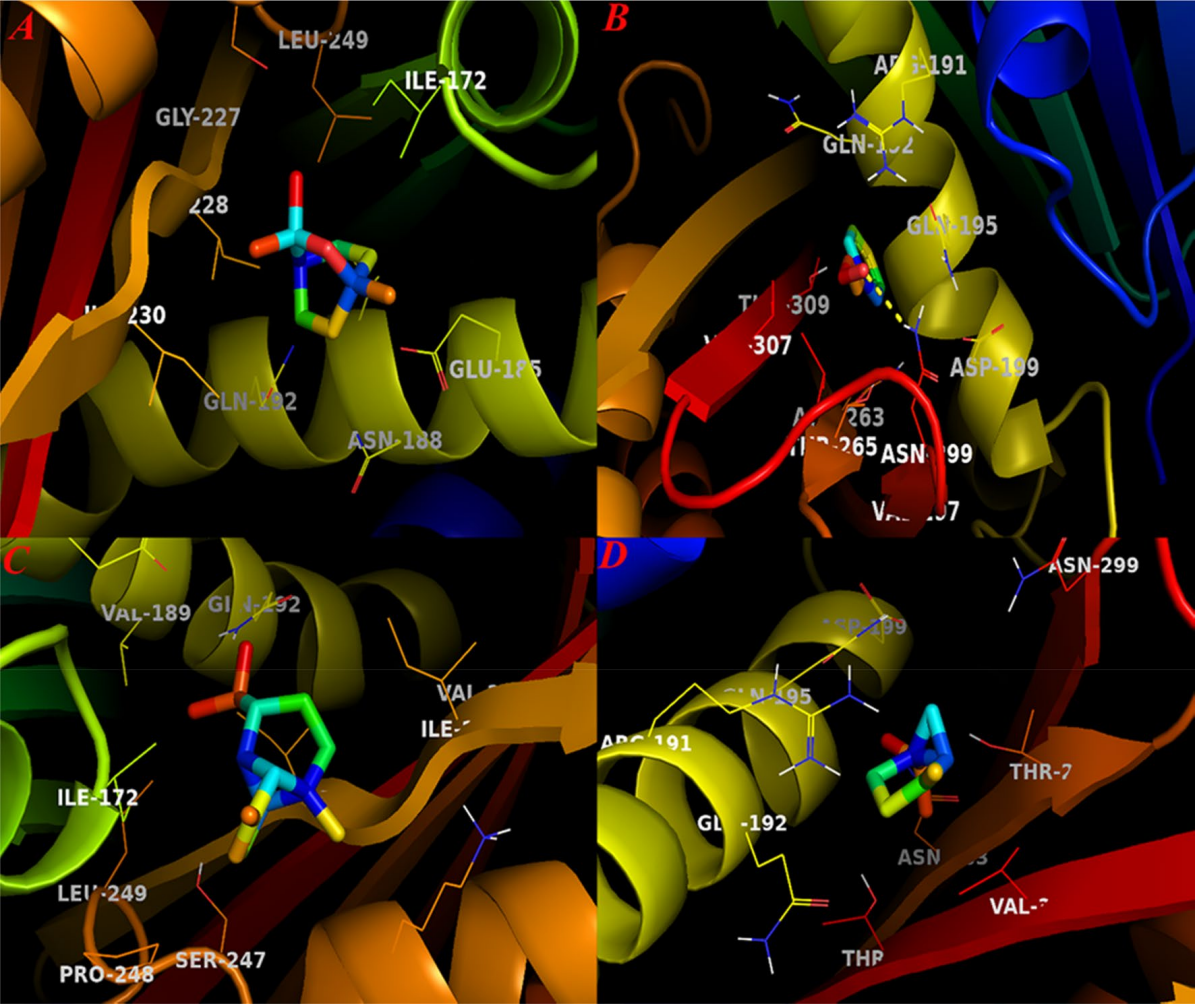
Graphical illustration docked compounds and FtsZ active site. **A** 1,8-cineole; **B** E-cinnamaldehyde; **C** Copaene and **D** Sabinene

FtsZ protein was another receptor that used for docking analysis. Haydon et al. (2008) reported that residues including Ile172, Val173, Phe183, Gln192, Arg191, Ile228, Leu302 and Val307 are critical amino acids in FtsZ active site. According to their results, potential inhibitors can bind to this active site to disrupt the normal functionality of this protein. Our results showed that *E*-cinnamaldehyde possessed the best docking energy (− 6.1 kcal mol^−1^) for this active site and formed an observable H-bond. Sabinene was another metabolite that lodged inside this active site and possessed docking energy equals to − 5.0 kcal mol^−1^. Other compounds also interacted with FtsZ active site by gaining docking energies ranged from − 3.5 to − 5.0 kcal mol^−1^, respectively. Figure 5 represents graphical illustration of four docked compounds into FtsZ active site.

**Fig. 6:**
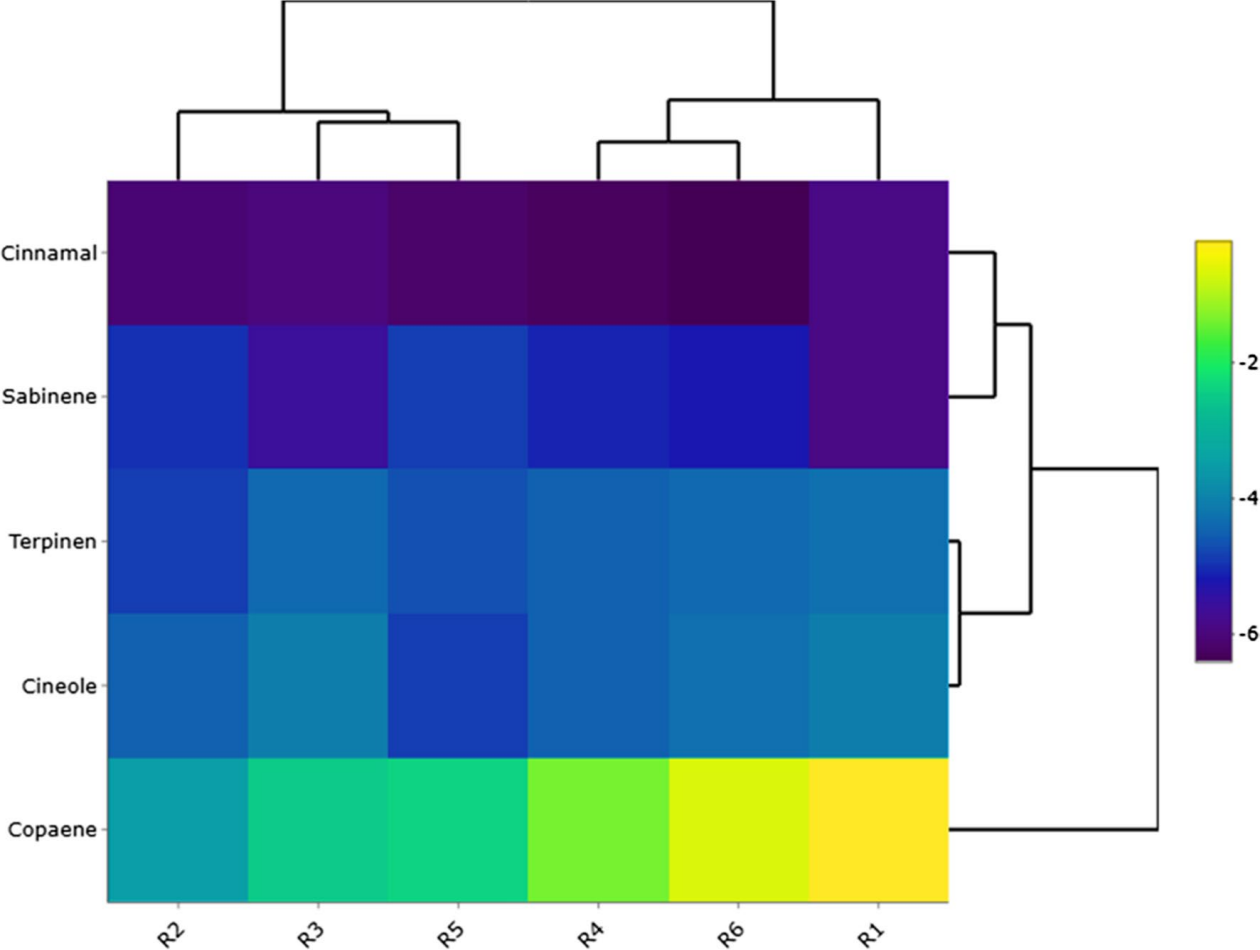
The correlation of docking energies between ligands and target receptors (R1 = Bap1; R2 = FabH; R3 = FtsZ; R4 = MerB; R5 = RbmC and R6 = RbmA)

For other receptors, the studied metabolites showed similar binding energies and *E*-cinnamaldehyde has built H-bond with all active sites (Additional file 1). These types of interactions suggested that individual substances of EOs may select different targets for showing their biological properties. Although further high-quality computational and experimental assays should be performed on these compounds to reveal their mode of action, the current results partially prove their potency to disrupt the formation of biofilm among studied infectious strains. According to Fig. 6, Copaene showed lowest binding energies for receptor 1, 4 and 6, respectively. At all, this compound was not able to form strong chemical bonds within the target active sites because of its unique chemical backbone. In contrast, *E*-cinnamaldehyde unravelled higher interaction tendency to approach the catalytic residues of target active sites. For other metabolites, similar binding energies for conducted docking analyses were observed.

**Fig. 7:**
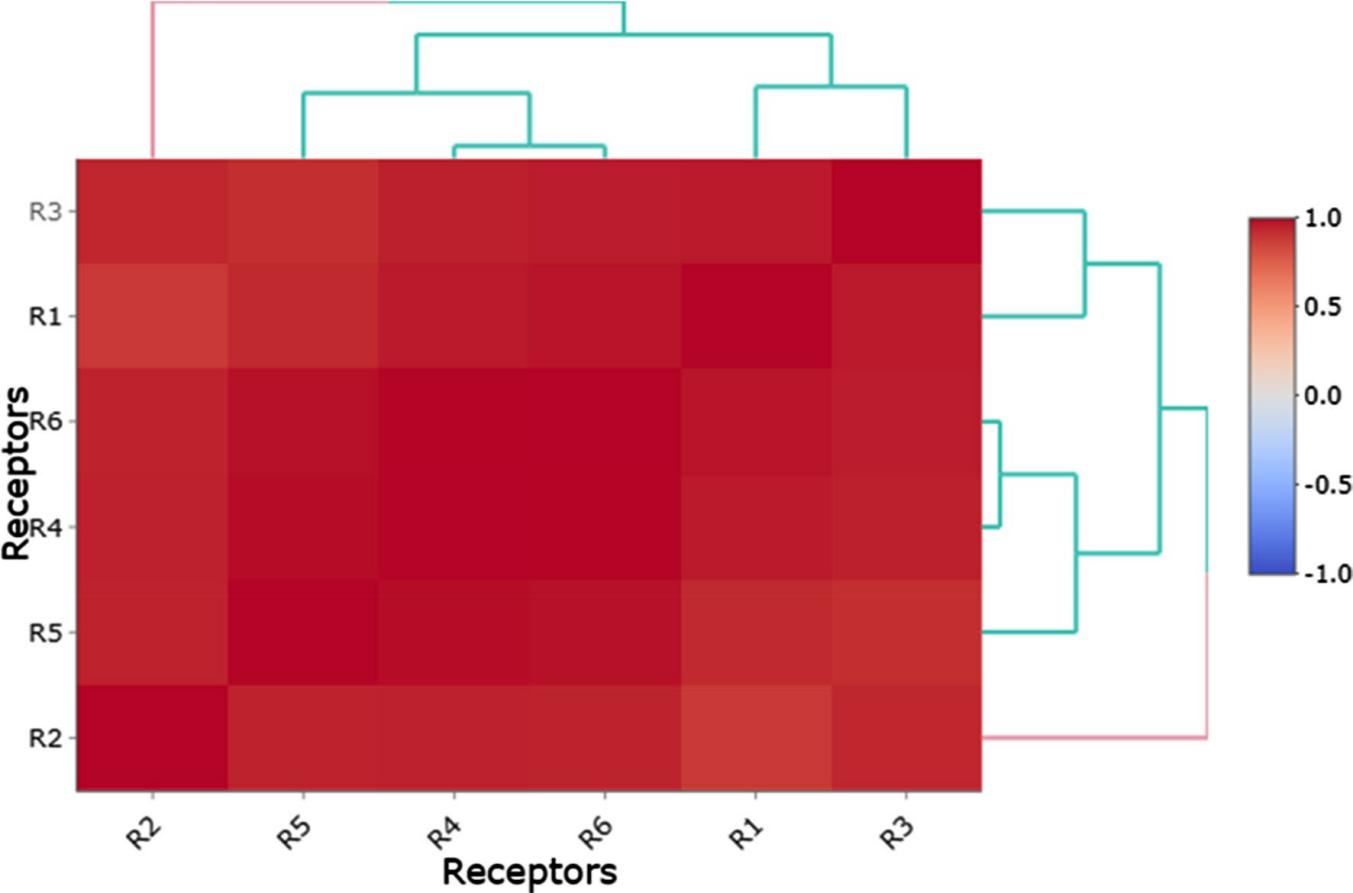
Correlation of docking energies between studied active sites and affinity of their cavity to interact with target ligands. (R1 = Bap1; R2 = FabH; R3 = FtsZ; R4 = MerB; R5 = RbmC and R6 = RbmA)

Based upon docking binding energies, the tendency of target receptors to interact with the docked compounds was investigated, and the results unraveled that receptors 4, 5 and 6 were the most favorable receptors to absorb the scaffold of ligands into their active site. Therefore, the conformational structure of active sites is a critical parameter that may change the interaction power between ligands and receptors. According to Rasouli et al. study (2020), to determine the type of interaction between ligands and active site residues, the most important factor is the variability of ligands backbones that could strongly affect the affinity of ligands to lodge into active site cavity. In their study, the authors determined that the differences between binding energies for docked compounds were due to structural changes of functional groups attached to the main scaffold of investigated plant metabolites. Here, our results also were in line with that study and the conformational size of docked active sites and chemical backbone of docked ligands determined the calculated binding energies. The following figure also illustrates the correlation of calculated binding energies for each receptor during molecular docking simulation (Fig. 7).

## Discussion

Today, finding an excellent and practical strategy to deter the possible growth of bacterial strains through inhibition of biofilm formation using various methods received a considerable attention from both academia and food industries. Among the applied methods for inhibition of biofilm formation, using natural products may provide lots of benefits for target industries because of availability of natural sources and their cheap cost. The most favorable technique that has presently been utilized is to apply EOs for this purpose. It seems quite clear that EOs are including considerable volume of purified secondary metabolites that can enter bacterial communities or cells to interact with a variety of molecular targets, leading to gradual inhibition of bacterial growth (Kerekes et al. 2019; Zhang et al. 2018).

As we shown in experimental assays, CiEO exhibited stronger antibacterial effect against both *E. coli* and *B. subtilis* (with lower MIC value of 0.08 and 0.31%v/v, respectively) compared with CaEO with MIC value of 2.5 (%v/v) against both bacteria. Interestingly, the growing body of evidence suggests that the *C. cassia* EO and its major constituent, i.e., cinnamaldehyde unravel active antimicrobials potential with a broad-spectrum activity (Chang et al. 2001; de Oliveira et al. 2012; Gill and Holley 2004; Ooi et al. 2006; Oussalah et al. 2007). Here, antibacterial activity of CiEO against *E. coli* was higher than *B. subtilis*. This result was consistent with the results by Fei et al. (2011) that reported CiEO showed higher antibacterial effect against *E. coli* (MIC value of 0.1 µl/ml) than *B. subtilis* (MIC value of 0.2 µl/ml) (Fei et al. 2011). Whereas CaEO had lower antibacterial effect against both of Gram-negative and Gram-positive bacteria with MIC value of 2.5 (%v/v) compared to CiEO. Asghar et al (2017) reported MIC values of CaEO in range of 5–10 (%v/v) against *S. typhimurium*, *S. aureus*, *S. mutans* and *C. albicans* bacteria strains (Asghar et al. 2017).

The GC/MS results showed, more susceptibility of *E. coli* to antimicrobial activity of CiEO than *B. subtilis* is because of E-cinnamaldehyde as a main component of CiEO. Trans-cinnamaldehyde (E-cinnamaldehyde) can inhibit the growth of *E. coli* and *S. typhimirium* without decomposing the outer membrane or depleting intracellular ATP and can also gain access to the periplasm and deeper portions of the cell (Helander et al. 1997, 1998; Nazzaro et al. 2013).Whereas the CaEO is composed of a considerable volume of monoterpenes. Various studies reported that monoterpenes are biologically active with moderate to high antibacterial profile against both Gram-positive and negative bacteria (Trombetta et al. 2005). The mechanisms underlying the antibacterial activity of monoterpenes is depending on variation of their chemical backbone and functional groups attached to their structure (Trombetta et al. 2005). Accordingly, the combination of EOs enriched with monoterpenes with other types of EOs comprised organic constituents may boost the strength of antibacterial activity of fused EOs. In this regard, as detailed in Table 2, synergistic and additive antibacterial effects for Ci/Ca EOs mixtures against *B. subtilis* and *E. coli* isolates were observed. To best of our knowledge, there is no study on the antimicrobial activities of the Ci/Ca EOs mixture against *E. coli* and *B. subtilis*: The published studies have focused on the antimicrobial activity of combinations between EO mixtures against *E. coli* (Fahimi et al. 2015; Semeniuc et al. 2017), thyme/cinnamon EO mixtures against *B. subtilis* and *E. coli* (Semeniuc et al. 2017). Among these combinations, only thyme/oregano (Stojković et al. 2013), thyme/myrtle (Sadiki et al. 2014), thyme/cinnamon (Kon and Rai 2012) and thyme/peppermint EO mixtures (Fahimi et al. 2015) unraveled a synergistic effect. The other combinations have displayed indifferent, additive, and antagonistic effects. In an interesting study, EOs synergistic effect on cell deformation, rupture and lysis of the membrane have been observed for *B. subtilis* cells treated with the combination of oregano oil and bergamot oil at MIC value while an incomplete and deformed shape observed in *E. coli* cells treated with the combination of oregano oil and basil oil at MIC value (Lv et al. 2011).

Normally, Gram-negative bacteria are more resistant than Gram-positive bacteria because of their characteristic structure of outer membrane serves as an additional barrier making them more resistant to lipophilic molecules and diffusion of the constituents of the EOs into the bacterial cell more difficult (Burt 2004; Mann et al. 2000; Nazzaro et al. 2013). However, in our study biofilm of *B. subtilis* was more resistant to Ci/Ca EOs than biofilm of *E. coli*. In this regard, studies reported that the Gram-negative bacteria biofilm (*E. coli* O157: H7) was more sensitive to antibacterial effect of carvacrol and eugenol compared to the Gram-positive bacteria biofilm (*L. monocytogenes*) (Perez-Conesa et al. 2006). Similar studies on *E. coli* and *L. monocytogenes* bacteria biofilms, the cells of Gram-positive *L. monocytogenes* were more resistant to *C. cassia* EO and cinnamaldehyde than the cells of the Gram-negative *E. coli* (de Oliveira et al. 2012). The antibacterial mechanisms of action of EOs and/or their components are closely related with their chemical compositions which can have a single target or multiple targets of their antibacterial activity. In this regard, EOs could have different mechanisms of action against Gram-positive and Gram-negative bacteria (Nazzaro et al. 2013). In our study, 1,8-cineole (with the highest amount of 49.10%) and terpinen-4-ol (with low amount of 2.15%) were the major components of CaEO. According to Kotan (Kotan et al. 2007)*,*lower antibacterial and antibiofilm activities of CaEO at MIC value of 2.5% than cinnamon may be related to higher content of 1,8-cineole and lower content of terpinen-4-ol. Singh et al. (2008) observed strong antibacterial activity for CaEO against *S. aureus*, *B. cereus* and *E. coli* at 3000 ppm by the agar well diffusion method. They reported α-terpinyl acetate (44.3%), 1,8-cineole (10.7%), α-terpineol (9.8%) and linalool (8.6%) as major components in which, in comparison to what was observed in our study, α-terpineol and linalool (oxygenated monoterpenes with considerable antibacterial activity) were in higher amount and 1,8-cineole (oxygenated monoterpene with no antibacterial activity) was in lower amount (Singh et al. 2008). This confirmed low antimicrobial activity of CaEO than CiEO in our study that it may be because of low amounts of α-terpineol and linalool in cardamom. Additionally, the higher antibiofilm potential of CiEO and its combination with CaEO against *E. coli* and *B. subtilis* is closely related to their chemical compositions, the action and the presence of certain or principal EO compounds. Terpenes and especially oxygenated monoterpenes consist a major proportion of cardamom EO and are the dominant groups of chemical constituents in the cardamom EO (Table 4). Oxygenated monoterpenes as widespread components of EOs are a class of terpenes that oxygenated (Chrysargyris et al. 2020). On screening of antibacterial activities of 21 oxygenated monoterpenes, linalool, nerol, α terpineol, fenchol, terpinen-4-ol showed antibacterial activity against a wide range of bacteria. In contrast to these compounds, no inhibitory activity was observed for camphor and 1,8-cineole against all tested bacteria (Kotan et al. 2007). Well-known oxygenated monoterpenes (e.g., citral, geraniol, linalool, menthol, and thymol) as the main components of distinct essential oils, alter the permeability of the cell by penetrating through fatty acyl chains of membrane lipid bilayers, disrupt lipid packing and change the fluidity of the cell membrane (Sánchez et al. 2004).

The mechanism underlying antibiofilm activity EOs is mainly not understood well but current predictions postulated that inhibition of enzymes involved in the formation of biofilm may be supported the purposed hypotheses. According to the review of the literature, several matrix proteins including RbmA, Bap1 and RbmC are key factors in the development and initiation of biofilm among bacterial strains. Among these proteins, RbmA-a 26 kDa matrix protein-displays a unique content to modulate the rugosity and structure of bacterial biofilm (Fong et al. 2006; Fong and Yildiz 2015). Mutants that have no coding sequence of *RbmA* gene could not produce strong and rigid biofilms; therefore, this case may indicate that the strength of bacterial biofilm is tightly depending on the regular expression of this protein (Fong et al. 2006). Additionally, the RbmA protein is involved in the formation of sessile biofilms and paly a quintessential role in the cell–cell and cell–surface interaction of bacterial community (Maestre-Reyna et al. 2013). Our docking simulation indicated that the identified metabolites among the studied plants have potential tendency to interact with crystallographic structure of biofilm matrix proteins through which may show their inhibitory profile. Among docked compounds, as detailed in result section, studied metabolites showed almost similar binding energies for hypothetical targets while cinnamaldehyde was displayed a better docking energy to interact with RbmA and C receptors, respectively (Figs. 6, 7).

The accumulating body of evidence reported that individual constituents of medical plants EOs are green alternatives of sodium hypochlorite and quaternary ammonium agents to prevent bacterial biofilms (Espina et al. 2015). E-Cinnamaldehyde or trans-Cinnamaldehyde was the most abundant moiety that identified within CiEO and studies reported that the antibacterial/antibiofilm activity of CiEO is associated with this secondary metabolite (Firmino et al. 2018). One molecular mechanism that has been considered for antibacterial activity of trans-cinnamaldehyde is to interact with ESBL enzymes (Dhara and Tripathi 2013). These categories of enzymes are mainly involved in the various resistance process within bacterial cell (Dhara and Tripathi 2013). Nakamura et al. (2019) recently reported that ESBL protein–protein interactions may be involved in the bacterial biofilm formation, though further studies should be performed to prove this hypothesis (Nakamura et al. 2019). Indeed, other studies also reported that that antibacterial activity of cinnamaldehyde is correlated with its potential to prevent fatty acid biosynthesis within bacterial cells. In a coupled experimental/computational study, Song et al. reported that cinnamaldehyde derivatives could deter the activity of β-Ketoacyl-acyl carrier protein (ACP) synthase III enzyme that critically is involved in bacterial fatty acid biosynthesis (Song et al. 2014). In this regard, Dubois-Brissonnet et al. (2016) reported that bacterial biofilm formation is associated with an increase in bacterial membrane saturated fatty acids. Therefore, it can be concluded that CiEO individual components may disrupt such processes within bacterial cells to overcome the growth of infectious bacterial communities. Accordingly, our computational assay also showed that cinnamaldehyde also showed high tendency to interact with FabH active site residues. The interaction of this metabolite and FabH active site may provide extra evidence for determination of molecular mode of action of CiEO compounds against bacterial cells (Fig. 7).

By and large, CaEO despite of having high portion of oxygenated monoterpenes exhibited lower antibiofilm activity than CiEO. Docking results for individual constituents of these two EOs also showed that their metabolites could interact with several different bacterial targets with moderate to low binding energies. The variability of those binding energies may prove this idea that the antibacterial activity of these EOs metabolites may follow up different actions to disturb the sustainable growth of bacterial community. Because of low amount of important oxygenated monoterpenes with antibacterial activity (such as geraniol, linalool, terpinen-4-ol, α terpineol) in CaEO, the lower antibiofilm property can be interpretated. All in all, the antimicrobial effects of essential oils are a result of the interaction between all components of the essential oils and no single compound effects (Delaquis and Stanich 2004; Lis‐Balchin and Deans 1997; Mourey and Canillac 2002). The use of a specific EO compound alone is not effective enough for inhibition of biofilm growth (Sandasi et al. 2008). Therefore, the inhibition activity of CaEO on bacterial growth was probably due to the interaction of the main compounds with other oxygenated monoterpenes and the high inhibitory activity of CiEO against bacterial biofilm might be related to high amount of E-Cinnamaldehyde constituent. Therefore, such EO compounds (well-known oxygenated monoterpenes and E-Cinnamaldehyde) and Ci/Ca EOs combination might be an effective green alternative against bacterial cell attachment and biofilm formation as observed here.

## Supplementary Information

**Additional file 1.** Docking outputs for 1hn9 and docked ligands. Docking outputs for 2wxy and docked compounds.

## Data Availability

The authors declare that all the data is embedded in the manuscript.

## Abbreviations

CaEO: Cardamom EO
CiEO: Cinnamon EO
Ci/Ca Eos: Cinnamon and cardamom EOs
CV: Crystal violet
EO: Essential oil
GC/MS: Gas chromatography-mass spectrometry
FIC: Fractional inhibitory concentration
FICI: FIC index
MIC: Minimum inhibitory concentration
MTT: (3-(4,5-Dimethyl-2-thiazolyl)-2,5-diphenyl-2H tetrazolium bromide)
PBS: Phosphate-buffered saline
XTT: (2,3-Bis(2-methoxy-4-nitro-5-sulfophenyl)-2H-tetrazolium-5-carboxanilide
