# Supplementary material for: Decoding antibacterial and antibiofilm properties of cinnamon and cardamom essential oils: a combined molecular docking and experimental study
Source: AMB Express. 2021 Oct 26;11:143. doi: 10.1186/s13568-021-01305-6 (PMC8548479; doi:10.1186/s13568-021-01305-6)
Supplement: Supplementary file 1 — Additional file 1. Docking outputs for 1hn9 and docked ligands. Docking outputs for 2wxy and docked compounds. [file 13568_2021_1305_MOESM1_ESM.docx]

Docking outputs for 1hn9 and docked ligands


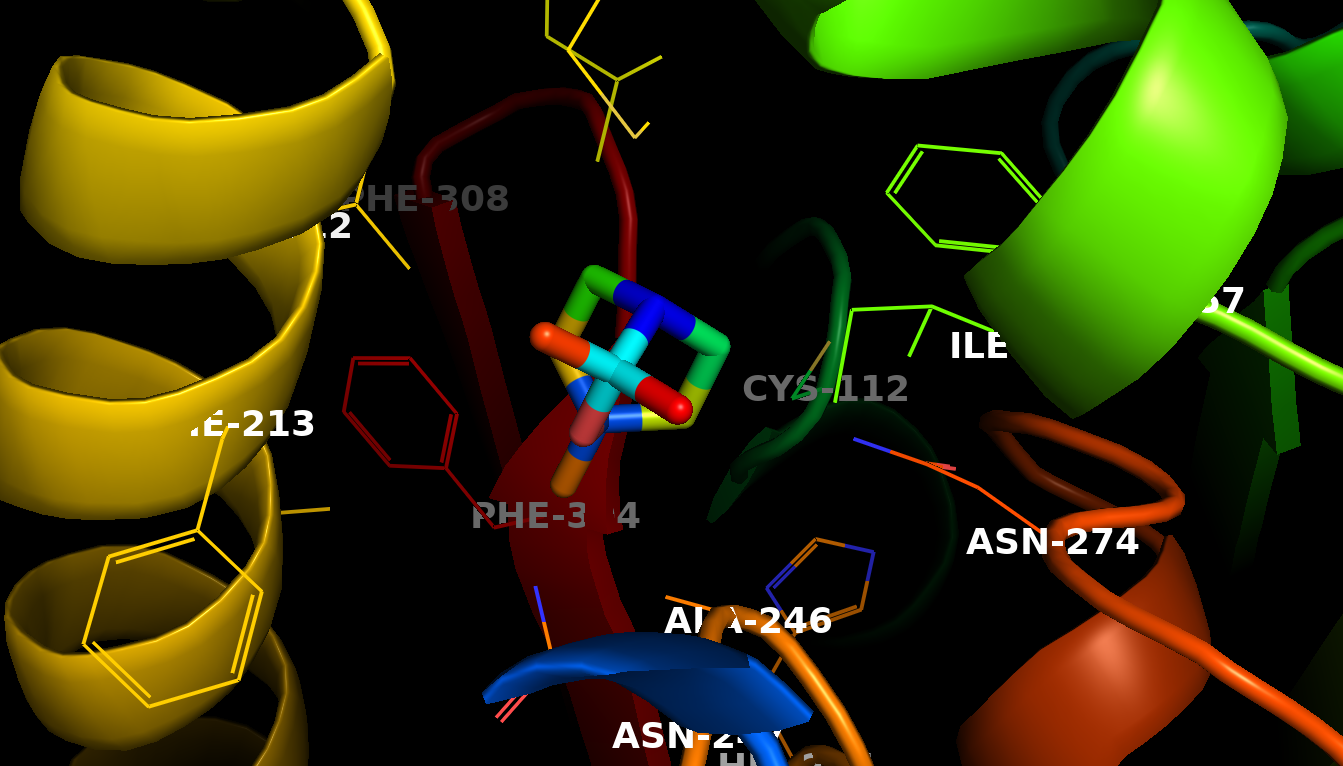


1,8-cineol


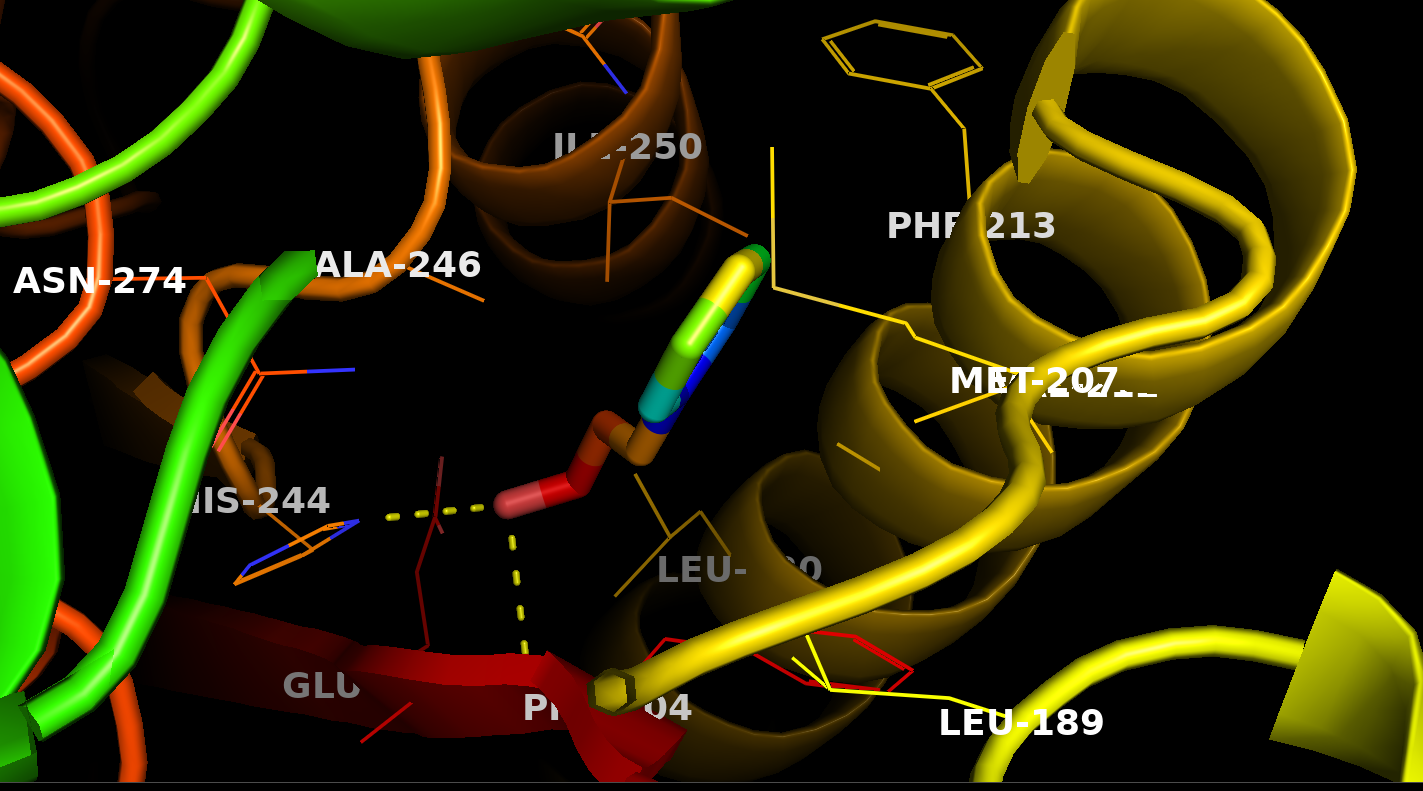


**trans**-**cinnamaldehyde**

**
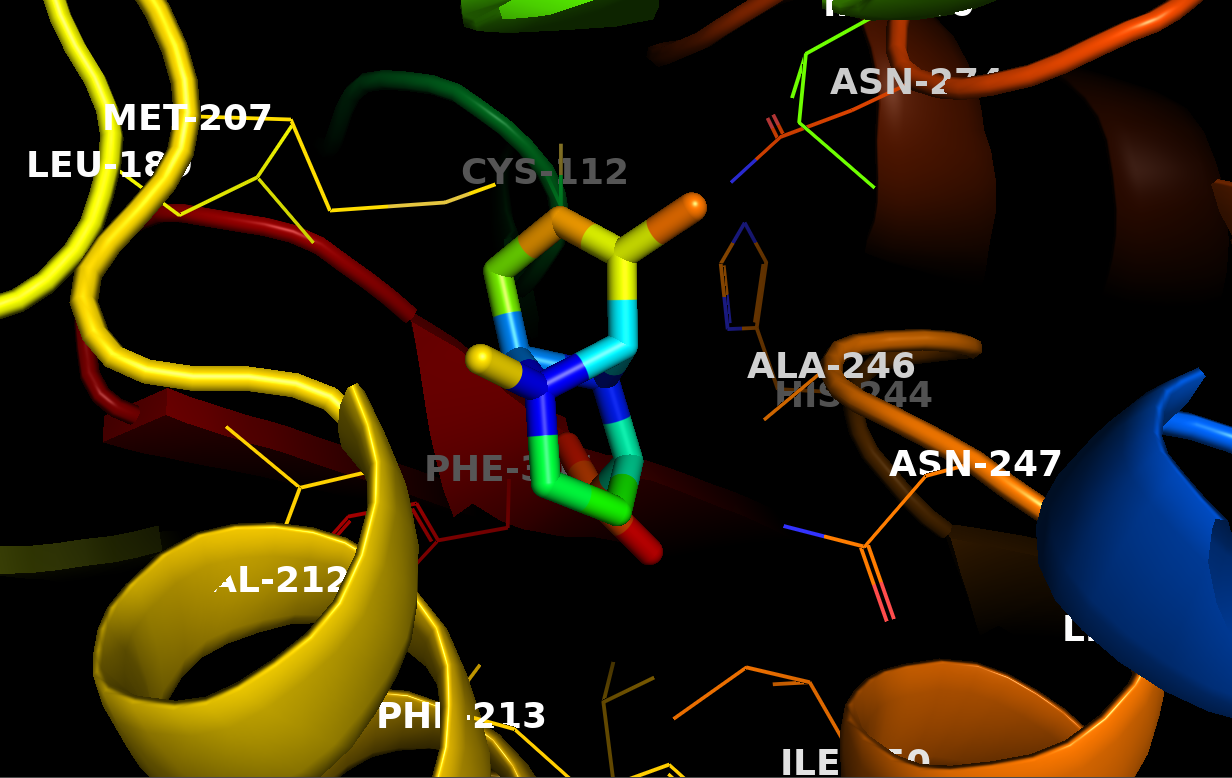
**

**Copaene**

**
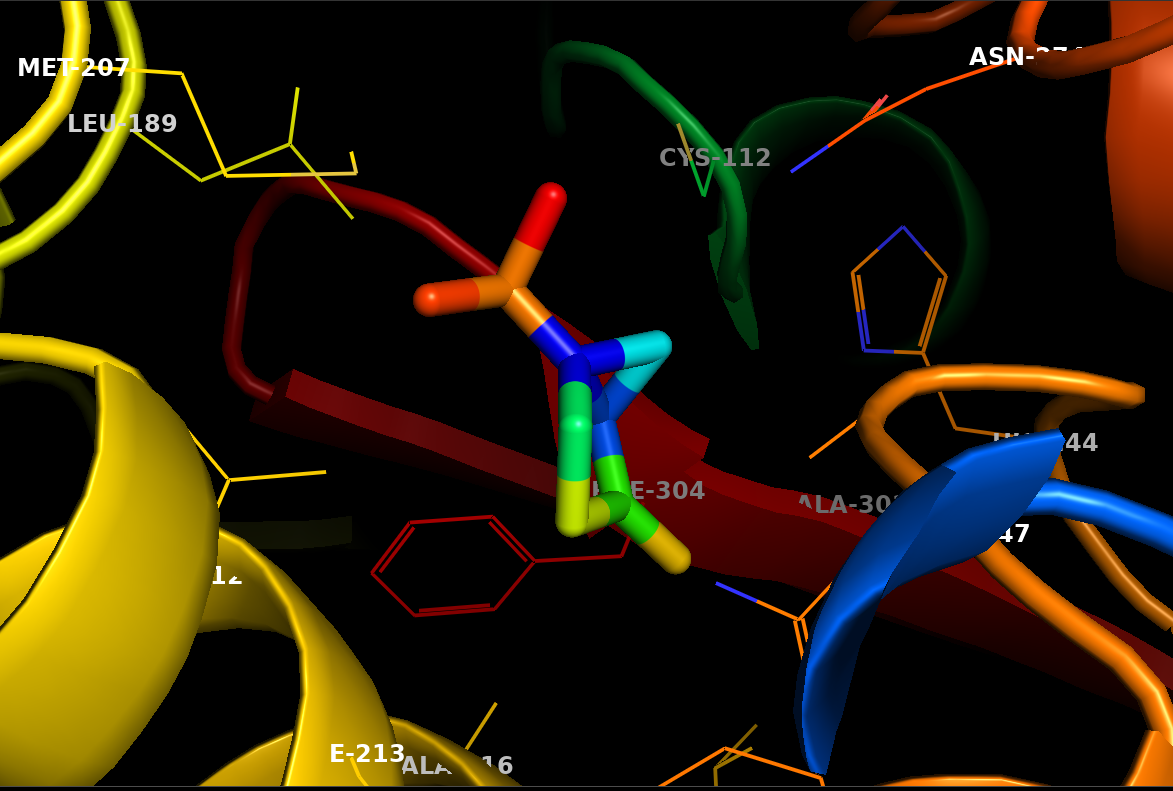
**

**Sabinene**

**
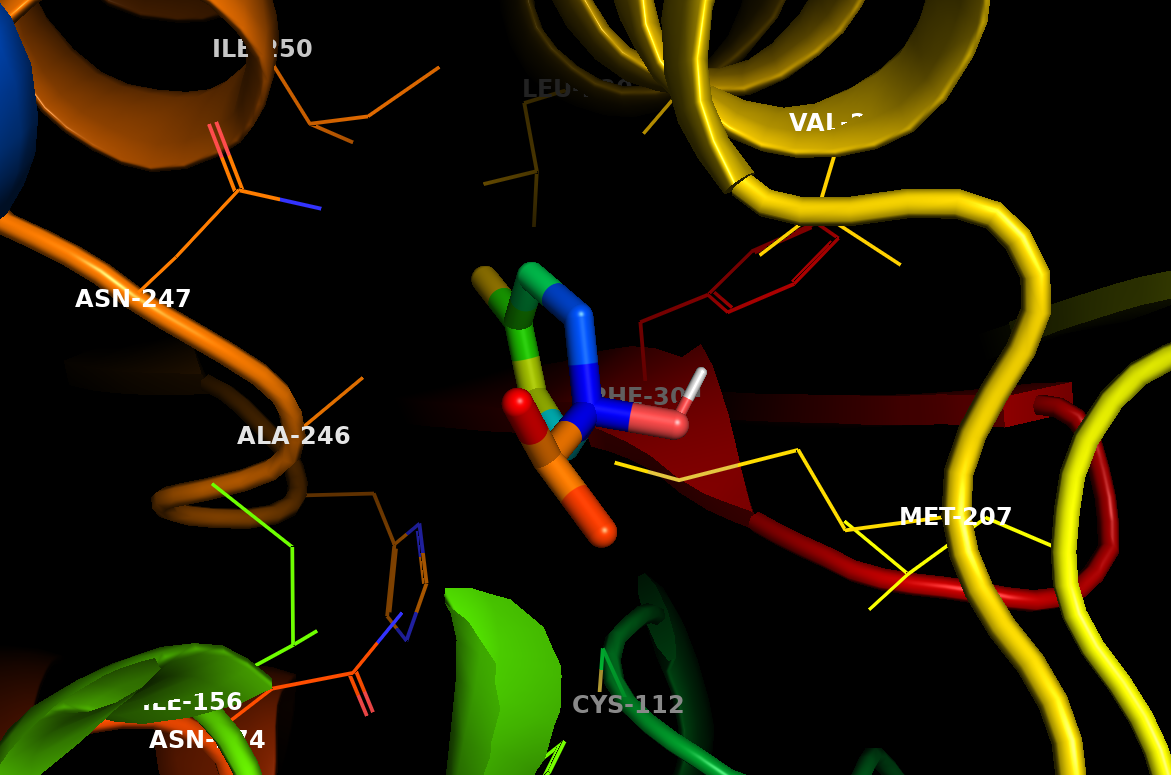
**

**Terpinen-4-ol**

Docking outputs for 2wxy and docked compounds


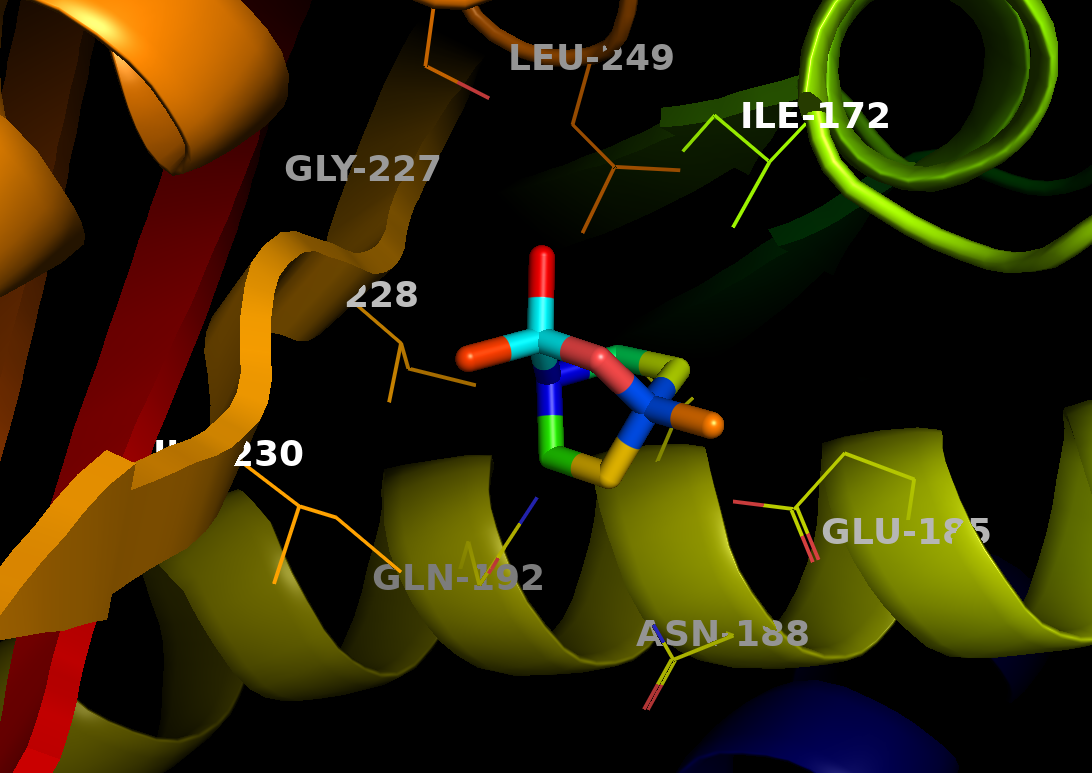


1,8-cineol


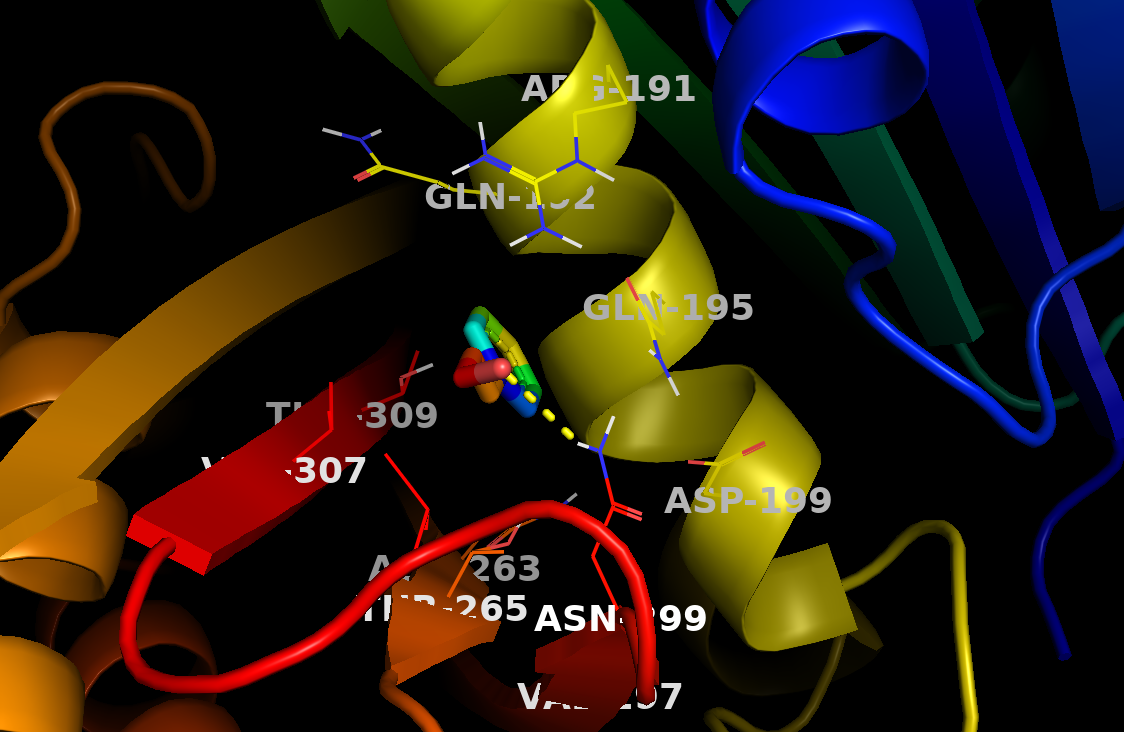


**trans**-**cinnamaldehyde**


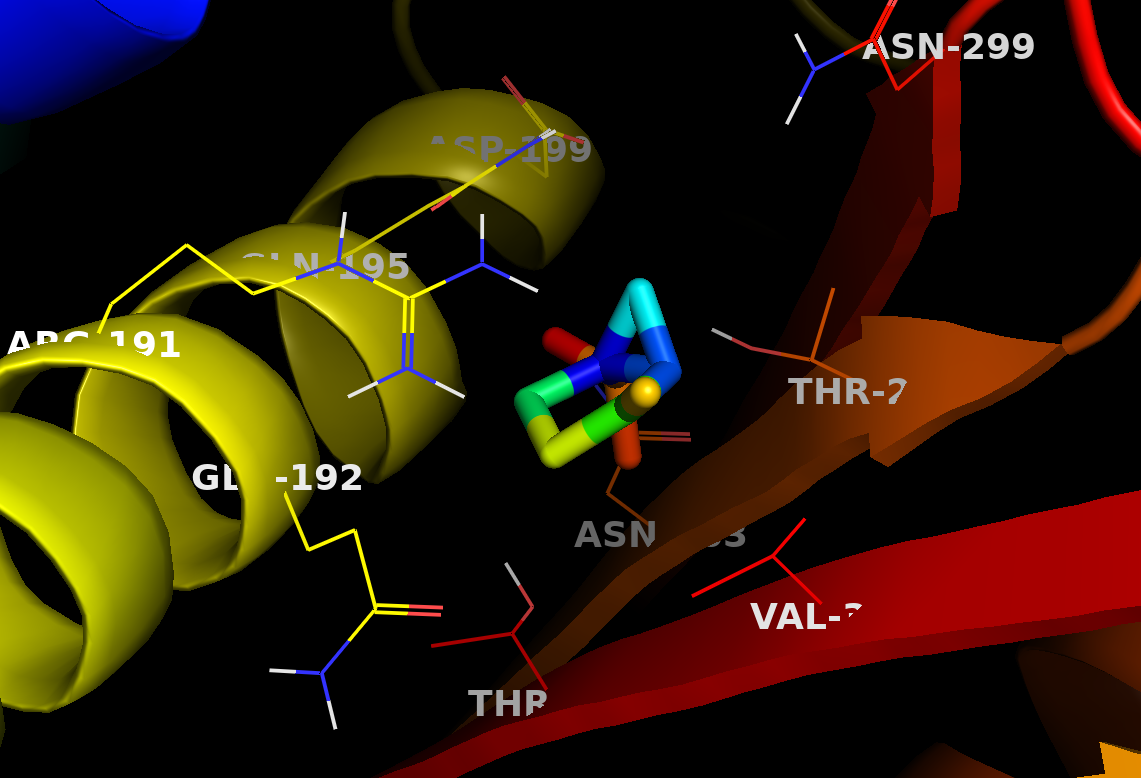


Sabinene


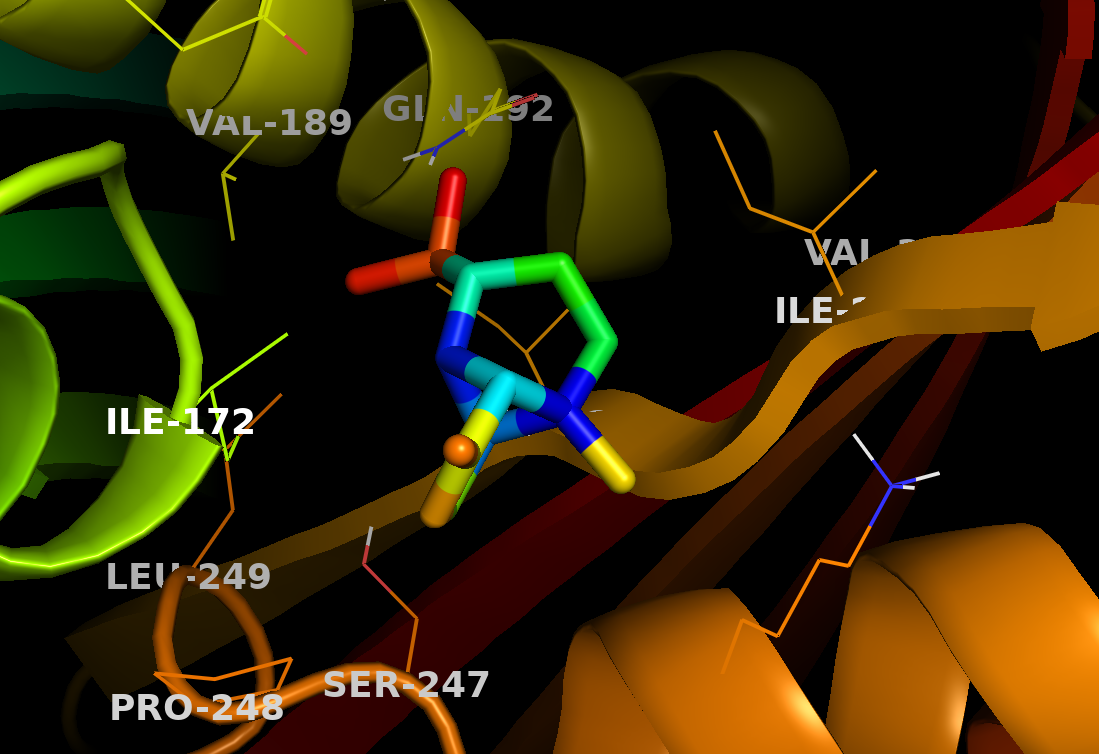


Copaene
